# Pregnant Smokers’ Experiences and Opinions of Techniques Aimed to Address Barriers and Facilitators to Smoking Cessation: A Qualitative Study

**DOI:** 10.3390/ijerph16152772

**Published:** 2019-08-02

**Authors:** Libby Fergie, Tim Coleman, Michael Ussher, Sue Cooper, Katarzyna A Campbell

**Affiliations:** 1 Division of Primary Care, School of Medicine, University of Nottingham, Nottingham NG7 2RD, UK; 2 Population Health Research Institute, St George’s University of London, London SW17 0RE, UK; 3 Institute for Social Marketing, University of Stirling, Stirling FK9 4LA, UK

**Keywords:** smoking in pregnancy, smoking cessation, behavior change techniques, qualitative research, interviews

## Abstract

Pregnant women experience certain barriers and facilitators (B&Fs) when trying to quit smoking. This study aimed to elicit women’s views on techniques that could help overcome or enhance these. Semi-structured interviews were conducted with 12 pregnant women who had experience of smoking during pregnancy. Participants were prompted to discuss experiences of B&Fs and give suggestions of techniques that could address these appropriately. A thematic analysis was conducted using the one sheet of paper method. Four themes relating to suggested techniques were identified: accessing professional help, nicotine replacement therapy (NRT), distraction, and social interactions. Experiences of accessing professional help were generally positive, especially if there was a good rapport with, and easy access to a practitioner. Most women were aware of NRT, those who had used it reported both negative and positive experiences. Praise and encouragement from others towards cessation attempts appeared motivating; peer support groups were deemed useful. Women reported experiencing B&Fs which fell under four themes: influence of others, internal motivation, cues to smoke, and health. Overall, accessing professional support generated positive changes in smoking habits. Establishing ways of how to encourage more women to seek help and raising awareness of different types of support available would seem beneficial.

## 1. Introduction

Smoking during pregnancy poses substantial health risks for both the mother and baby. The mother’s risk of pregnancy complications such as placenta abruption, placenta previa [1], miscarriage, stillbirth, ectopic pregnancy, and premature labor are increased [2]. The consequences for the babies can be lifelong with their risks of low birth weight, birth defects such as cleft lip, asthma [2], cognitive impairments [3], diabetes [4], childhood malignancies [5,6] and becoming a smoker later in life being increased [7]. Continued smoking during pregnancy is a global health concern. A recent review of data from 174 countries found prevalence rates to be over 20% in 12 and over 10% in 29 countries [8]. Currently in England, 10.8% of women continue to smoke throughout their pregnancy which Public Health England aim to reduce to 6% or less by end 2022 [9]. As part of trying to reach this target, it is necessary to fully understand the reasons why women continue to smoke and what techniques could be helpful in supporting a successful quit attempt.

Pregnancy is a time when women are generally highly motivated to try and stop smoking [10], however, they may experience certain barriers, which can impinge on cessation attempts, or facilitators which can make quit attempts easier. Examples of barriers include partners’ continued smoking and social norms that encourage and normalize smoking whereas the desire to protect the baby from unnecessary harm can help facilitate a successful quit attempt [11,12,13].

Evidence shows that behavioral support interventions, comprised of behavioral change techniques, can help women overcome barriers or optimize facilitators and successfully achieve cessation during pregnancy [14,15]. There have been literature reviews [14,15,16] and work carried out with experts in the field of smoking in pregnancy, including health care professionals, stop smoking practitioners, academics and public health specialists to establish which techniques could be effective when used as part of a cessation support intervention [13]. Extensive qualitative work has also helped to establish pregnant women’s, partners and practitioners views on existing cessation support [11]. However, as there is little work exploring what techniques would be useful and acceptable from the women’s perspective, this study aimed to enhance this body of evidence, by gaining pregnant women’s opinions on techniques which they have found to be, or think could be, useful in helping overcome the barriers or enhancing the facilitators towards achieving cessation.

## 2. Materials and Methods

Ethical approval was granted by the East of Scotland Research Ethics Service REC 1. The Consolidated Criteria for Reporting Qualitative Research (COREQ) criteria [17] was used to inform the methods, analysis and write up of the study.

### 2.1. Recruitment

Potential participants were identified through antenatal clinics at two hospital trusts in the East Midlands, UK: Nottingham University Hospitals NHS Hospitals Trust (NUH) and Sherwood Forest Hospitals NHS Foundation Trust (SFHFT). In line with ethical approval, women were considered eligible if they were 18 years old or over, had an appropriate command of spoken English, ability to give informed consent, pregnant and currently smoking or recent quitter who had smoked during their pregnancy. The recruitment process at each clinic was agreed with the clinical lead. At the NUH clinics, potential participants were identified through the use of screening forms. At the SFHFT clinic, members of the midwifery team discussed the study, on a one to one basis, with women who were either current or ex-smokers. All eligible women who expressed an interest in taking part, received a telephone call from a researcher (L.F.; an author) inviting them to participate in an interview.

### 2.2. Semi-Structured Interviews

The interviews were conducted over the telephone by a female researcher (L.F.) who has a background in adult nursing and qualifications in Psychology (BSc) and Health Psychology (MSc), with a specific interest in behavior change. Previous experience of qualitative work include carrying out interviews and focus groups covering issues surrounding smoking and various long-term health conditions. The interviewer introduced herself as a researcher from the University of Nottingham. It was explained to the women that the interviewer was not a stop smoking practitioner and, as such, was unable to offer cessation advice, however, the purpose of the interview was to learn about their experiences of smoking during pregnancy with an aim to help enhance cessation support that is offered to pregnant clients in future. The women were informed that the interview could take up to 40 min, would be recorded and that any quotes from the interviews used in publications would be anonymized. If they agreed to be interviewed, they were asked to give verbal consent to take part both before and after the recording started.

### 2.3. Interview Guide

A semi-structured interview template was used to guide the discussions. There was patient and public involvement (PPI) input into the design and content of all study documents, the methods of recruitment and the interview topic guide to ensure they would be acceptable for the women. Conversations were initiated by asking about the pregnancy in general and how the women felt about their smoking. The women were then asked about their attitudes towards cessation and experience of any quit attempts. In order to form a basis for discussions surrounding techniques that could be useful in assisting a quit attempt, the women were then prompted to talk about anything they had encountered that made trying to quit easier or more difficult. Women were then asked about any techniques they had tried and how effective they had found them in terms of overcoming any difficulties or enhancing the positive influences they had experienced. They were also prompted to give suggestions of any other techniques that they thought might be helpful. If they had accessed any professional cessation support, they were asked about their experience and opinions surrounding this. At the end of the interview, they were given the opportunity to discuss anything they felt was relevant or important to add that had not been covered during the interview. The women were sent a £ 20 high-street voucher as compensation for their time. Each participant was interviewed only once. All telephone interviews were digitally recorded.

### 2.4. Analysis

The recordings were transcribed verbatim. L.F. read the transcripts on completion and corrected any misinterpretations of the recordings. NVivo qualitative data analysis software (Version 11, QSR International Pty Ltd., Melbourne, Australia) was used to store the transcripts and support the analysis. In order to comprehensively capture all data from the women’s suggestions on techniques that could address the barriers and facilitators (B&Fs), and identify patterns emerging from these, a thematic analysis was carried out using the one sheet of paper (OSOP) method [18], guided by the six phases of qualitative analysis as described by Braun and Clarke [19]. This involves writing all the different issues raised in relation to each code, with extracts and participant unique identifiers (IDs), on one page and establishing from this how such issues can be clustered together to form comprehensive themes [18]. This was done initially by one researcher (L.F.) then reviewed by a second (K.A.C). Any disagreements were resolved through discussion and amendments to the overarching themes and coding were made accordingly. In order to reach data saturation, which was considered to be the point that no new codes were being identified in the data set [20], it was intended that a minimum of ten interviews would be conducted. Although the focus of the study was not on B&Fs, the same analysis was performed on the data relating to these in order to establish if women had experienced any that had not been identified through previous work. In order to make more sense on the data for the suggested techniques and understand which barrier or facilitator they were aimed to address, the analysis on the data relating to B&Fs was carried out first. Findings from the B&Fs analysis were compared with previous findings [11,12,13] to establish if there were any dissimilarities.

## 3. Results

During recruitment, a total of 33 eligible women, who expressed an interest in taking part, were identified. All 33 were contacted, 12 agreed to be interviewed. Eleven of the interviewees were white British, one was black British. Ten had partners, none were married. All participants were resident in Nottinghamshire. Interviewees’ ages ranged from 19 years–40 years (m = 27.7, SD = 6.7) and they were between 17 and 38 weeks pregnant (m = 30.4, SD = 5.7). Seven were currently smoking but had cut down; three of whom had stopped briefly, four had quit and one had switched exclusively to using an e-cigarette (see Table 1). All but one explicitly expressed that they would like to quit. The duration of the interviews ranged from 10.5 min to 35.5 min (m = 18.4, SD = 7.1). 

### 3.1. Analysis on the Barriers and Facilitators

This section reports a summary of the main findings from the analysis on the data relating to B&Fs. Four main themes were identified: influence of others, internal motivation, cues to smoke and health, examples of which are given in Table 2. There were a range of significant others who could influence the women’s smoking habits, both in a personal or professional capacity, however the smoking patterns of partners, family and friends appeared to have a strong influence on whether the women were tempted to continue to smoke. Having a partner who continued to smoke tended to tempt the women to want to smoke too, however, if the partner was supportive of their attempts to quit or cut down, the women found this to be particularly helpful regardless of the partners’ smoking habits. Overall, being pregnant was frequently mentioned as being an important internal motivator to want to quit, whereas stress was often mentioned as a cue to smoke and attributed to continued smoking. There were three sub-categories relating to health that all seemed important in terms of influencing the women’s motivation towards cutting down or quitting: the women’s own health in general, pregnancy specific issues and the health of the baby. The factors relating to health were more commonly facilitators towards quitting rather than barriers. In relation to previous literature, no new barriers or facilitators were identified.

### 3.2. Techniques that Could Help Overcome Barriers or Enhance Facilitators towards Cessation

**Figure 1:**
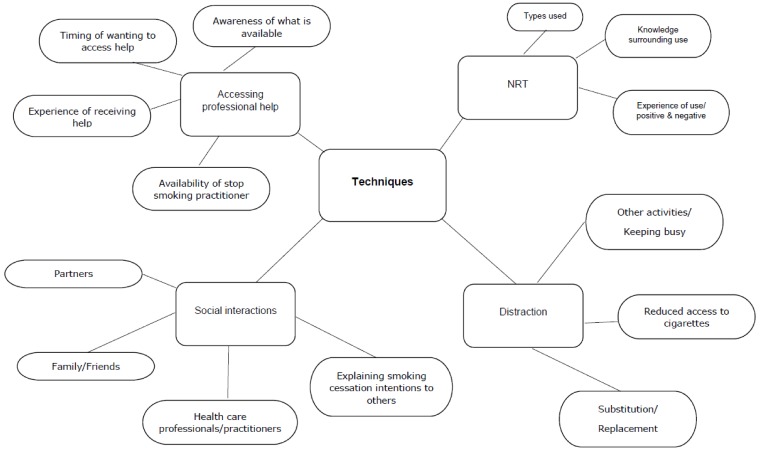
Themes, sub-themes from the analysis of suggestions for techniques that could support cessation.

This section gives a full overview of the findings from the analysis on the data from suggestions for techniques the woman thought were or could be useful in overcoming barriers and enhancing facilitators towards quitting. Four themes were identified: accessing professional help, nicotine replacement therapy (NRT), distraction from smoking and social interactions in relation to cessation. A diagrammatic representation of the themes and sub-themes from the OSOP is displayed in Figure 1.

#### 3.2.1. Accessing Professional Help

Accessing a stop smoking practitioner, which related to B&Fs that fell under the theme of ‘influence of others’, appeared to help from the perspective of having someone to talk things through with, receiving reassurance about their experience throughout the cessation process and overcoming barriers such as cues to relapse:

“I think it’s important to have somebody, like from a service, you know, so they can talk you through it and say it is normal … because I was worried, you know, I was still having cravings like up til now and she says ‘no that’s normal, sometimes it takes people a couple of weeks, sometimes it takes people a couple of months, everybody’s different’, but to answer those questions and just to have somebody, you know, keeping check, you know, and see how you’re doing, it helps.”

Participant 2.

Overall, women reported positive experiences in regards to accessing professional help. This tended to be especially for women who believed they had an open, honest and non-judgmental relationship with their stop smoking practitioner. For example, this type of relationship appeared to help one woman overcome a relapse:

“…[W]hen I walk in her room we have a good laugh and a good giggle and we have a good chat, she isn’t one of these that just gets straight down to business and lets you blow in the meter and sends you on your way and just asks you questions and questions and questions, she makes sure that you’re alright in yourself.”

Participant 4.

“I did admit it to my adviser, I had a fag, I had one … I had that fall back and we talked through it and we got through it.”

Participant 4.

Good availability and immediacy of access to a stop smoking practitioner when required appeared important for the women. Some had more positive experiences when the practitioner was readily available, however, one woman felt that if she’d been able to have immediate access to an advisor it might have helped prevent her return to smoking.

“Well I get an advisor so, you know, if I’m having a bad day and I need to phone her, I could … I can just phone her up at the club.”

Participant 2.

“I weren’t due to see her til the following Tuesday and over the weekend I felt really low … you know because I’d been doing it [not smoking] for that long and it made me feel really, really down and I just had to have a smoke … if I’d have seen her sooner I think that might have helped.”

Participant 11.

In addition to having a good rapport and being able to easily access a stop smoking practitioner, women reported that when practitioners used visual aids this had an impact on them. This mainly related to B&Fs that fell under both the ‘health’ and ‘internal motivation’ themes:

“… she told me about the placenta as well, the placenta of a smoker, she showed us some pictures … and I was shocked … a normal healthy placenta and then somebody who smokes, it looks like it’s dead, you know, so that made a big impact, I think that would help anyone pregnant that wanted to stop smoking seeing those pictures, definitely.”

Participant 2.

Not all the women had accessed professional help, although those who had not were aware that this was available. The reasons for not engaging with stop smoking services varied from not knowing what type of help they offered, to thinking that they did not smoke enough to need professional support to quit. Some women stated that they would prefer to delay seeking professional help until the baby was born.

“[Interviewer: what sort of support so you think will be helpful] I honestly don’t know … I don’t know what sort of things are out there.”

Participant 6.

“I didn’t smoke enough when I got pregnant to need that help necessarily.”

Participant 1.

“I think once I’ve had her I’m going to get, like help.”

Participant 12.

#### 3.2.2. Nicotine Replacement Therapy

NRT was a cessation aid often mentioned in the interviews. Discussions surrounding its use were mainly initiated from the discussions on the B&Fs relating to ‘influence of others’. The women’s knowledge and perceptions surrounding NRT appeared generally to be influenced by both health care professionals and people from their close social network. Most of the women appeared to be aware of, and some explicitly reported being offered, a range of NRT products:

“They [hospital-based stop smoking practitioners] just came out and offered nicotine replacement.”

Participant 7.

“I could have stopped [smoking] using patches, gum, the spray, the lozenges, the sticks, most of the aids that are available she’s [stop smoking practitioner] been able to offer.”

Participant 4.

In relation to being useful in supporting quit attempts, overall the women who had used NRT found it to be beneficial, although some reported side effects. Other women expressed the opinion that certain products were not an adequate replacement for cigarettes.

“I had the Nicorette patch and I had the inhalator. Because you’re supposed to choose two products … That was good. I stopped three full days as well as in the morning, which was the worst one, you know what I mean, or after eating.”

Participant 11.

“I chose the chewing gum [Interviewer: did that help?] Yeah, it has.”

Participant 4.

“I had the gum which made me have heartburn and the patches which made me itch.”

Participant 5.

“… [B]ecause she [stop smoking practitioner] was saying there’s chewing gums and whatever available. But for me, it’s not necessarily about replacing the nicotine. Obviously that would affect it … but for me, it was the habit of the hand to the mouth. So I needed that substitute of fag, if that makes sense?”

Participant 8.

Similarly with e-cigarettes, although there were positive experiences of use, with one woman having switched solely to using them, some women also commented on negative aspects and concerns of the use of these devices during pregnancy.

“E-cigarettes are meant to be a substitute [but] it’s not exactly the same. I know with the cigarette you get a complete release from stress when you smoke one, whereas with the E-cig, I think it’s more like the placebo effect … I still don’t get the same satisfaction that I would do from a cigarette.”

Participant 10.

“I have thought about that [trying an e-cigarette] but I don’t know if they’re worse with all the studies … I just know in the past, on the news they say they can be more harmful when pregnant.”

Participant 11.

#### 3.2.3. Distraction from Smoking

Techniques that involved distraction from smoking were perceived as being helpful specifically in relation to barriers that fell under the theme ‘cues to smoke’; for example, negative emotion, being in a situation where they would normally smoke, or times when they would normally have their most wanted cigarette. Distraction techniques mainly consisted of keeping busy with activities other than smoking which involved using their hands. A few mentioned physical exercise, which they said mainly relieved stress, but was also suggested as a way to take their minds off smoking:

“Like first thing in the morning, I used to wake up and have a fag straightaway in bed. That was difficult at first … I overcome that by, you know, like instead of smoking, doing the kids’ sandwiches…”

Participant 2.

“I was thinking about … getting a gym membership as obviously exercise releases the stress … if you wake up and you put smoking at the back of your head, and, especially because going to the gym is to do with being healthy … it makes me not want to smoke.”

Participant 10.

A similar technique, relating to distraction, was for women to reduce their access to cigarettes. Regardless of whether this was done intentionally or unintentionally, by themselves or by a supportive other, it seemed generally to be effective:

“…[H]e [partner] supports me because he’ll hide his tobacco and fags so I can’t find them if I wanted one anyway.”

Participant 4.

“I think any sort of illness or like hospitalisation where I was away from it [smoking]…makes you realise that you’ve gone a period of time without it so you realise you’re not dependent upon it.”

Participant 3.

#### 3.2.4. Social Interactions in Relation to Smoking and Cessation Attempts

In relation to the B&Fs that fell under the theme ‘influence of others’, the influence of partners, family/friends, health care professionals and peers had was reported as being a barrier in some instances and a facilitator in others with the women showing a tendency to following these others’ smoking habits. For the women whose partner, family or friends continued to smoke, some found it helpful to use techniques including removing themselves from situations where they would be tempted to smoke with them:

“It’s getting him [partner] to not smoke around me … sometimes I’ll go upstairs because he’s having a cigarette.”

Participant 6.

“… [M]y Mum and my sister, they smoke a hell of a lot, so there’s always going to be someone like sparking up … so I just have to stay away from them, you know what I mean, for a bit.”

Participant 11.

Although having a partner who continued to smoke tended to tempt the women to want to smoke too, if the partner was supportive of her attempts to quit or cut down, the women found this to be particularly helpful regardless of the partners’ smoking habits. Having good support from family members, especially those who decided to quit too also seemed valuable and helped enhance facilitators relating to ‘internal motivation’ in particular:

“Yeah, he [partner] was helpful, he was very like cheering me on and ‘you’re doing really well’ and everything, yeah that helped.”

Participant 11. (partner was still smoking)

“… [It] was more my Mum and my Grandma, because they used to smoke and they both made a pact together to quit, and they went onto e-cigarettes, and they kind of supported me and helped me to do the transition the way they did.”

Participant 10.

Women did not find it helpful if others, including health care professionals and family members, simply told them to stop without offering any form of support to encourage or help them to stop:

“They [midwives] just keep telling me to stop … it winds me up a little and makes me worse [with smoking].”

Participant 7.

“I’ve got certain family members that are ‘well it’s about time you stopped smoking’ … ‘well I’ve been telling you for years you should stop’ … Whereas it’s not the help that you need. That’s not help. That puts more pressure on you to say ‘well you’re not supporting me, you want me to stop smoking but you’re telling me that I should have done it years ago, not praising me for actually being say seven days without a cigarette.”

Participant 4.

Peer support groups appeared to be favoured by women who had participated in them and these were also suggested by women when asked for other ideas of what may help. The specific group activities mentioned included sharing experiences they found useful and using distraction techniques:

“Or maybe I’d join a group with other people going through the same thing, to hear what they’re doing to cope … and they might be doing things that are helping them that I haven’t tried yet, so that would be good to explore other options.”

Participant 10.

“I have had help from the non-smoking group at the hospital, the midwife put me through to them, so they’ve helped me with the patches and I go to pregnancy club once a week … we do discuss the no smoking thing but we like make friendship bracelets or do pregnancy exercises …”

Participant 2.

## 4. Discussion

This study reports pregnant women’s experiences and opinions on techniques they have found to be, or think may be, useful in helping achieve smoking cessation during pregnancy. Having good accessibility to a stop smoking practitioner, especially who the women had a good working relationship with, was deemed helpful, although some women, who had not accessed support were less aware of how this might help them. Overall, amongst those who had used NRT most found it helpful, however some reported side effects. There appeared to be differences in opinion on the sufficiency of NRT products and e-cigarettes as a substitute for cigarettes. Concerns were also expressed over perceived safety of e-cigarette use during pregnancy. Using techniques to distract from smoking appeared useful, especially in a situation or at a time the women would be most likely to have a cigarette. Women were motivated by encouragement and praise towards cessation attempts from significant others, regardless of whether these persons continued to smoke. Peer support groups were also deemed helpful. In relation to barriers and facilitators, no additional ones from those identified prior to this study [11,12,13] were found. Being pregnant appeared to be a good facilitator towards quitting, stress was often mentioned as a barrier.

### 4.1. Strengths and Weaknesses

Establishing women’s views on techniques that may be useful gave a deeper understanding on what might or might not be helpful when used as part of an intervention aimed to support cessation during pregnancy. This information will be of particular use for intervention designers and practitioners delivering cessation support interventions. A strength of this study is its unique approach whereby women were asked to generate discussions around techniques they had tried, or thought may be useful. This meant we were less likely to restrict discussions to techniques proposed by the researcher and so potentially miss important techniques; it also gave a good overview of how the techniques can be operationalized in a way that was acceptable to the women.

There were some limitations to this study. Smoking in pregnancy is a sensitive and often stigmatized topic and, as such, pregnant women tend to be reluctant to fully disclose their smoking behaviors [21], especially as there is increasing pressure on women to quit both from health care professionals and society in general [22]. It is possible that the responses given may not have been a true reflection of smoking patterns for reasons such as fear of being judged negatively or being put under further pressure to quit. In order to try and overcome this, we adopted the telephone interview method which, although has been criticized for compromising rapport building and missing non-verbal cues, it is also thought to allow participants the opportunity to openly disclose sensitive material in a non-intimating and anonymous manner [23]. As it was explained at the beginning of the interviews that the researcher was not qualified to give cessation advice and that we only aimed to gather their opinions and experiences to help women in the future, it is hoped that any apprehensions about being actively encouraged to quit would have been allayed at an early stage.

The reluctance to disclose smoking status and perceived stigma surrounding smoking in pregnancy can also impact on participation rates [21]. In this study, of those identified as being eligible, less than half agreed to be interviewed, which could be a result of this reluctance. It is also possible that the women who agreed to be interviewed were more willing to discuss their smoking status with health care professionals and as such would have been more likely to have accessed cessation support. As the women interviewed were from one region of England, where rates of smoking at time of delivery were higher than the 10.8% national average (NUH clinics: 17.8%, SFHFT clinic: 24.9%) [24], with levels of deprivation being relatively high compared with other regions of England, UK [25], these findings may be more relevant to women within areas of similar demographics.

However, the minimum recommendation of ten interviews in order to reach data saturation [20] was exceeded and no new codes were identifiable by the time the twelfth interview was conducted which meant we could be confident that all relevant themes were identified. Furthermore, using the OSOP method [18] in the analysis allowed all aspects within a theme to be displayed together, with any nuances being captured at a glance, thereby allowing for a comprehensive and rich analysis [26].

### 4.2. Discussion of Findings

In line with evidence which shows smokers to be more likely to quit if they seek professional help [27], for the women in this study who had accessed professional help, their experience of this and smoking outcomes were generally positive. It would therefore seem beneficial for midwives and other antenatal health care professionals to reassure the women, that regardless of how much they smoke, accessing professional help could increase their chances of quitting and be beneficial to themselves and the baby.

In general, there were positive experiences of both NRT and e-cigarette use amongst the women. However, similar to the beliefs of some health care professionals who are not qualified stop smoking practitioners [28], a few of the women reported safety concerns surrounding the use of e-cigarettes during pregnancy. The fact that e-cigarettes were not perceived to be a substantial enough replacement, particularly relating to stress relief, was also mentioned. Likewise, NRT products were viewed as an insufficient replacement to some as they did not replace the hand-to-mouth action of cigarette smoking. Also, amongst the women who had used NRT there were some reports of side effects such as heartburn and itching. Understanding that women have some adverse experiences with NRT during pregnancy and ensuring that the women know all options that are available to them would also be beneficial, especially as preferences appear to vary considerably between individuals. Providing clearer information on the safety of NRT and other nicotine delivery systems use during pregnancy to women and professionals who provide antenatal care, but are not stop smoking practitioners could also help.

It is known that women’s smoking habits are often influenced by their immediate social environment [29,30], for example, one qualitative study highlighted that women justified continued smoking due to ‘living in a smoking world’ [31]. From this it has been recommended that women’s wider social setting should be taken into account when offering cessation support [29]. In this study it was identified that the women’s smoking habits tended to follow those of their partners, families and friends. This could act as a barrier or a facilitator depending on whether these others continued to smoke or had cut down or quit during the duration of their pregnancy. In addition to involving significant others in the quit attempt, it would be helpful for the stop smoking practitioner to be aware of the type of influence these others have on the women, in terms of smoking habits, to be able to address this accordingly [32]. As the women found it encouraging if they were given praise, despite the significant other’s smoking status, it would help to prompt people in the woman’s close network to use this technique.

The women often mentioned stress as being a reason for continued smoking. As stress is a known predictor of continued smoking and relapse amongst quitters [33,34], it has been recommended that it is acknowledged by those designing or delivering interventions [35,36,37]. Despite this, recognizing and dealing appropriately with stress by stop smoking practitioners, when offering support to pregnant women, has been found to be done inconsistently across National Health Service (NHS) cessation services [35]. Addressing issues surrounding stress in interventions for pregnant clients would therefore seem an element of support that warrants priority. As part of this, it could be encouraging for practitioners to highlight to women that their stress levels and any depressive symptoms they may have are likely to decrease, with general mental wellbeing being more likely to improve when they quit [38,39]. It would also be advisable to dispel any myths, such as the stress due to the process of stopping being worse for the baby than continuing to smoke, which was a concern for one woman in this study. The women also mentioned activities such as physical exercise and breathing exercises as distraction techniques which have also been shown to help reduce stress, support cessation and reduce cravings and withdrawal symptoms amongst smokers in general [40,41,42]. Encouraging engagement in such activities may be acceptable to women, if deemed appropriate by the practitioner.

### 4.3. Further Work

Gaining deeper insight into the reasons why some pregnant women do not access stop smoking services would be beneficial. Assumptions can be made as to what the possible underlying causes could be. For example, it may be that by putting off accessing help until after the baby is born, the women are using avoidance tactics due to fear of being stigmatized if they are seen to be accessing these services while they are visibly pregnant [43,44]. Work of this type would help to give an indication of any extra support women in this category might need and also what aspects of existing support could be changed to encourage more women to seek help. In relation to accessibility to practitioners, although there are organizational and funding related restrictions on the type of service stop smoking practitioners can provide [13,28], it is recommended that practitioners are flexible in their availability for pregnant clients, which includes being available between scheduled appointments, as much as restrictions allow. Otherwise it may be helpful if the women are signposted to additional options for support, for example online helplines.

The finding that women perceive attending peer support groups as beneficial has been reflected in findings of studies looking at smokers in the general population [45] and in particular in low-income female smokers [46]. It does however differ from a study of pregnant women’s preferences in terms of types of support which showed individual rather than group based support to be more desirable, possibly due to feelings of discomfort or shame in disclosing or discussing their smoking habits with others whilst pregnant [47]. More work could help establish in what context group support could be helpful in a way that is acceptable to the women. This would seem important especially as pregnant women who smoke often lack adequate social support towards achieving cessation [48].

## 5. Conclusions

Having a good, non-judgmental working relationship with a practitioner, who the women had good accessibility to, especially in times of greater need for support, was deemed useful. The techniques that were perceived to be particularly useful included distraction, praise and peer support groups. Better advice on the overall use of NRT and other nicotine delivery devices, including safety of use in pregnancy and what products are available could also be beneficial. Findings from this study reiterate that being pregnant is a strong motivator to stop smoking however certain influences, in particular stress, can override this and result in continued smoking. Women had a tendency to follow the smoking habits of significant others which is an aspect that warrants consideration whilst designing or delivering cessation support interventions.

## Figures and Tables

**Table 1:** Smoking status of participants at time of interview.

Participant Identifier (ID)	Age	Smoking Status
Participant 1	19	Had quit
Participant 2	36	Had quit
Participant 3	27	Had quit
Participant 4	40	Had quit
Participant 5	20	Still smoking but had cut down
Participant 6	27	Still smoking but had cut down
Participant 7	33	Still smoking but had cut down (managed to quit briefly)
Participant 8	29	Still smoking but had cut down (managed to quit briefly)
Participant 9	20	Still smoking but had cut down
Participant 10	22	Switched to solely using e-cigarettes
Participant 11	32	Still smoking but had cut down (managed to quit briefly)
Participant 12	27	Still smoking but had cut down

**Table 2:** Examples of barriers and facilitators experienced by participants.

Influence of Others	
B:	Participant 9: “Well quite a few of my family smoke, so, I mean I’d say that tempts me to smoke when they’re around and stuff”
F:	Participant 1: ““I started to feel funny after having like a fag … so I turned to my partner when I had enough of it and I was like ‘just don’t let me have one even if I beg for it’ and he didn’t so here I am”
Internal motivation	
B:	Participant 6: “… the second I found out [about being pregnant] I did want to quit, but I just couldn’t do it this time … I just couldn’t stop”
F:	Participant 10: “the only reason why I quit was because I was pregnant, so I mean, if I hadn’t fallen pregnant, I’d probably still be smoking cigarettes now”
Cues to smoke	
B:	Participant 1: “I wasn’t pleased [about smoking] but I was like in a really stressful place”
F:	Participant 4: “But now sitting on a bus I’d get up and move seats just to move away from a smoker because I can’t stand the smell”
Health	
F:	Participant 4: “… because it’ll [staying quit] also help my health because I’ve got asthma and osteoarthritis as well, so it’s like if I stop smoking it helps with my health because it helps with my lungs and my asthma, but then it also helps if I’ve got more energy …” (Existing health conditions)
F:	Participant 3: “It was just that I kept being sick so I didn’t have the inclination to want to smoke just in case it made me worse” (Pregnancy related health issues)
F:	Participant 5: “If it put my children in danger … Like if my daughter stopped growing while she was inside me” (Babies’ health)

Barrier (B), Facilitator (F).

## Data Availability

All data are available on request from the corresponding author.
